# Meniscus gene expression profiling of inner and outer zone meniscus tissue compared to cartilage and passaged monolayer meniscus cells

**DOI:** 10.1038/s41598-024-78580-3

**Published:** 2024-11-09

**Authors:** Kaileen Fei, Benjamin D. Andress, A’nna M. Kelly, Dawn A. D. Chasse, Amy L. McNulty

**Affiliations:** 1 Department of Orthopaedic Surgery, Duke University School of Medicine, DUMC Box 3093, Durham, NC 27710 USA; 2 Department of Pathology, Duke University School of Medicine, DUMC Box 3093, Durham, NC 27710 USA; 3 Department of Biomedical Engineering, Duke University, DUMC Box 3093, Durham, NC 27710 USA

**Keywords:** Meniscus, Cartilage, Transcriptomics, Dedifferentiation, Monolayer, Tissue engineering, Biomedical engineering, Cell biology

## Abstract

Meniscus injuries are common and while surgical strategies have improved, there is a need for alternative therapeutics to improve long-term outcomes and prevent post-traumatic osteoarthritis. Current research efforts in regenerative therapies and tissue engineering are hindered by a lack of understanding of meniscus cell biology and a poorly defined meniscus cell phenotype. This study utilized bulk RNA-sequencing to identify unique and overlapping transcriptomic profiles in cartilage, inner and outer zone meniscus tissue, and passaged inner and outer zone meniscus cells. The greatest transcriptomic differences were identified when comparing meniscus tissue to passaged monolayer cells (> 4,600 differentially expressed genes (DEGs)) and meniscus tissue to cartilage (> 3,100 DEGs). While zonal differences exist within the meniscus tissue (205 DEGs between inner and outer zone meniscus tissue), meniscus resident cells are more similar to each other than to either cartilage or passaged monolayer meniscus cells. Additionally, we identified and validated *LUM, PRRX1,* and *SNTB1* as potential markers for meniscus tissue and *ACTA2*, *TAGLN, SFRP2,* and *FSTL1* as novel markers for meniscus cell dedifferentiation. Our data contribute significantly to the current characterization of meniscus cells and provide an important foundation for future work in meniscus cell biology, regenerative medicine, and tissue engineering.

**Supplementary Information:**

The online version contains supplementary material available at 10.1038/s41598-024-78580-3.

## Introduction

The menisci are fibrocartilaginous tissues located in the knee joint and are essential for proper joint biomechanics. Meniscal injuries are one of the most common knee injuries and remain one of the most common indications for orthopaedic surgery^1^. However, not all meniscal lesions are amenable to surgical repair, and research is currently aimed towards investigating biomaterials and bioengineered tissues for improved meniscus healing^2,3^ or replacement of lost and damaged tissue^4,5^. While significant effort has been made to create meniscus tissue-engineered constructs that can functionally replace damaged tissue^6,7^, there remain significant gaps in knowledge surrounding meniscus cell biology. Overall, poor characterization of the specific meniscus cell transcriptome and limited understanding of the transcriptomic changes that occur due to monolayer culture expansion remain major hurdles facing the field of meniscus tissue engineering^8–12^.

It has long been appreciated that the meniscus structure and extracellular matrix (ECM) composition are phenotypically distinct from other musculoskeletal tissues^13,14^. The meniscus can be divided largely into two zones, the inner and outer zones, which differ in ECM composition, vascularity^2^, and resident cell morphology^7,15,16^. Clinically, the inner and outer zones are frequently referred to as white and red zones, respectively, due to the avascularity of the inner zone and the presence of a blood supply in the outer zone. While previous work has characterized the differential expression of ECM and catabolic genes^9,10,17^ and cell surface markers^11,18^ of inner and outer zone meniscus cells, a comprehensive evaluation of the transcriptomic profile of these zones is still needed. Therefore, despite the recognition that meniscus cells are a phenotypically distinct cell type, researchers in the field of meniscus cell biology are ultimately left defining meniscus cells in relation to more well-characterized cell types, commonly referring to outer zone cells as “fibroblast-like” and inner zone cells as “chondrocyte-like”^7,8,11^.

More recently, state-of-the-art genomic technologies have been used to examine the meniscus cell transcriptome, as it relates to embryonic meniscus development^19^, aging^20^, mechanotransduction^21,22^, and osteoarthritis^23,24^. While these studies have begun to elucidate the genetic signature of meniscus cells, there remains a need to fully characterize the zonal variation in meniscus cells, elucidate the transcriptomic differences between meniscus tissue and articular cartilage, and to further explore the underlying gene expression changes that contribute to dedifferentiation of meniscus cells during monolayer culture. In this study, RNA-sequencing was used to compare 1) cartilage to inner and outer zone meniscus tissue and 2) inner and outer zone meniscus tissue to passaged inner and outer zone monolayer meniscus cells.

## Results

**Fig. 1:**
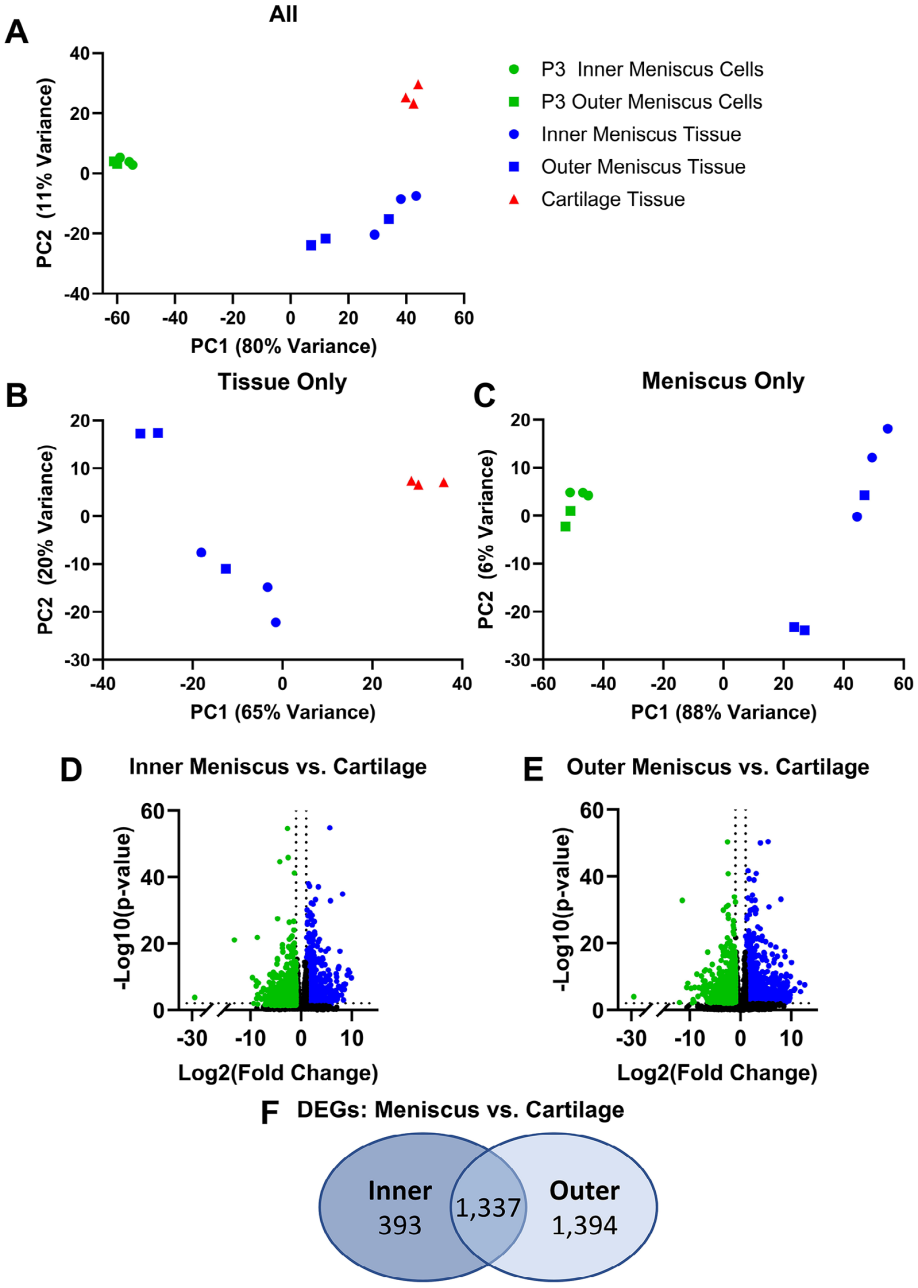
Comparison of Meniscus, Cartilage, and Passage 3 (P3) Meniscus Cells using Principal Component Analysis (PCA) and Differential Gene Expression Analysis. PCA comparing (**A**) all samples (cartilage, meniscus tissue, and P3 meniscus cells), (**B**) tissue samples (cartilage and meniscus tissue), or (**C**) meniscus samples (meniscus tissue and P3 meniscus cells). Shape corresponds to anatomic source (circle: inner zone meniscus, square: outer zone meniscus, triangle: cartilage), color denotes sample type (blue: meniscus tissue, green: P3 meniscus cells, red: cartilage tissue). Total variability accounted for by each principal component is indicated in the axis labels. Volcano plots show genes significantly differentially expressed between (**D**) inner zone meniscus tissue and cartilage, or (**E**) outer zone meniscus tissue and cartilage. Each data point is an individual gene. Dashed lines indicate cutoffs for significant genes (FDR adjusted p-value < 0.01 and a |base-2 log fold-change|> 1). Color denotes if the gene was significantly up-regulated (blue) or down-regulated (green) in meniscus tissue relative to cartilage, or not significantly different (black). (**F**) Number of significantly differentially expressed genes (DEGs) exclusive to or shared between inner and outer zone meniscus tissue comparisons to cartilage.

Bulk RNA-seq was performed on tissue explants taken from cartilage, inner and outer zone meniscus tissue, and passaged meniscus cells harvested from either the inner or outer zone. Principal component analysis (PCA) plots were used to visualize overarching transcriptomic differences and display a clear separation of meniscus tissue, cartilage, and passaged meniscus cells. When comparing all groups, the first principal component (PC1), accounting for 80% of variance, primarily separated tissue samples from passaged cells, while principal component 2 (PC2, 11% of variance) separated meniscus tissue from cartilage (Fig. 1A). When analyzing only tissue samples, PC1, which accounts for 65% of the variance, separated cartilage from meniscus tissue, while PC2 (20% of variance), separated meniscus tissue samples primarily by zone, albeit with one outer zone sample clustering closer to the inner zone samples (Fig. 1B). When comparing meniscus tissue to passaged meniscus cells, PC1 (88% of variance) separated tissue from monolayer cells, while PC2 (6% of variance) separated meniscus tissue samples, again mostly delineating inner from outer (Fig. 1C). Passaged cells from both inner and outer zones cluster closely together and were not separated by zone in these analyses (Fig. 1A,C).

Pairwise comparisons of DEGs between meniscus tissue and cartilage were performed to better quantify transcriptomic differences. A total of 1,730 genes were significantly differentially expressed (p < 0.01, |LogFC|> 1) between inner zone meniscus tissue and cartilage (Fig. 1D and Supplemental Dataset 1), while 2,731 DEGs were identified between outer zone tissue and cartilage (Fig. 1E and Supplemental Dataset 2). A total of 1,337 of the DEGs were shared between both inner and outer zone comparisons to cartilage. On the other hand, 393 DEGs were exclusive to the inner zone and cartilage comparison, while 1,394 unique DEGs were identified when comparing outer zone tissue to cartilage (Fig. 1F).

To create a cartilage-specific expression profile, genes were sorted by greatest copy number in cartilage and the list of the top 25 expressed genes with logFC > 1 and padj < 0.01 relative to inner (Table 1) and outer zone tissue (Table 2) was generated. When comparing cartilage to inner zone meniscus, differential expression of genes associated with the following were identified: ECM components, including several collagen isoforms (*COL9A2, COL16A1, COL9A3, COL3A1*), *SERPINH1*, which is a collagen chaperone^25^, and several glycoproteins (*CHI3L1*, *BGN, CILP2,* and *TNC*); signal transduction, including *GEM*, which is a regulator of chondrogenic differentiation^26^; transcriptional regulation, including *AEBP1*, which is a transcriptional repressor and is involved in collagen polymerization^27^; cytoplasmic proteins *NUCB1*, which is a calcium-binding protein, and *SQSTM1*, which is involved in bone remodeling; enzymes *NT5E*, which hydrolyzes 5′-AMP into adenosine and phosphate, and *CDO1*, which enables cysteine dioxygenase activity; growth factors *LTBP1*, which is critical for TGF-β activity^28^, and *NDUFA4L2*, which is involved in mitochondrial function; and *EEF2*, which is essential for protein synthesis. Of these top 25 expressed genes, the most highly differentially expressed genes in cartilage relative to inner zone meniscus were ECM genes *COL9A2* (logFC = 5.15) and *ABI3BP* (logFC = 4.64), and transcriptional regulator *AEBP1* (logFC = 3.91). Transcriptional differences in cartilage relative to outer zone meniscus tissue identified genes associated predominantly with the ECM, including *COL2A1, CILP2, SPARC, ACAN, COL3A1, FMOD, BGN, COMP,* and *PRELP,* which are well-established cartilage matrix components, and protein synthesis, with 10 of the genes encoding ribosomal proteins and two being eukaryotic translation elongation factors. There were also changes in genes associated with signal transduction, such as *LCN2*, which is a catabolic adipokine^29^, and transcriptional regulators, including *AEBP1*. The most highly differentially expressed genes in cartilage compared to outer zone meniscus were ECM genes *COL2A1* (logFC = 4.69), *CILP2* (logFC = 2.47) and *SPARC* (logFC = 2.46). Overall, eight of the top 25 highly expressed genes in cartilage were significantly differentially expressed relative to both inner and outer zone meniscus (*CILP2, SPARC, BGN, COL3A1, EEF2, LCN2, AEBP1, TPT1*).

**Table 1:** Top 25 expressed genes in cartilage compared to inner zone meniscus tissue.

Gene symbol	Gene name	LogFC	padj
Cytoplasmic Protein			
NUCB1	Nucleobindin 1	1.26	1.83E-06
SQSTM1	Sequestosome 1	1.02	1.75E-14
Enzyme			
NT5E	5'-Nucleotidase Ecto	1.73	4.09E-04
CDO1	Cysteine Dioxygenase Type 1	1.57	8.35E-04
Extracellular Matrix			
COL9A2	Collagen Type IX Alpha 2 Chain	5.15	3.41E-06
ABI3BP	ABI Family Member 3 Binding Protein	4.64	2.85E-28
COL16A1	Collagen Type XVI Alpha 1 Chain	3.31	3.89E-11
COL9A3	Collagen Type IX Alpha 3 Chain	2.88	3.20E-03
FBLN7	Fibulin 7	2.65	4.96E-06
EFEMP1	EGF Containing Fibulin Extracellular Matrix Protein 1	2.23	7.82E-03
TNC	Tenascin C	1.89	1.36E-03
CHI3L1	Chitinase 3 Like 1	1.86	6.38E-03
SPARC	Secreted Protein Acidic And Cysteine Rich	1.64	7.29E-04
BGN	Biglycan	1.54	3.91E-06
**CILP2**	**Cartilage Intermediate Layer Protein 2**	**1.48**	**2.36E-03**
SERPINH1	Serpin Family H Member 1	1.48	8.89E-10
COL3A1	Collagen Type III Alpha 1 Chain	1.20	7.34E-03
Growth Factor and Regulator			
LTBP1	Latent Transforming Growth Factor Beta Binding Protein 1	1.20	5.98E-05
NDUFA4L2	NDUFA4 Mitochondrial Complex Associated Like 2	1.86	1.13E-03
Protein Synthesis			
EEF2	Eukaryotic Translation Elongation Factor 2	1.13	1.76E-07
Signal Transduction			
GEM	GTP Binding Protein Overexpressed In Skeletal Muscle	2.61	1.54E-07
LCN2	Lipocalin 2	1.77	1.24E-03
Transcriptional Regulation			
**AEBP1**	**AE Binding Protein 1**	**3.91**	**1.25E-09**
TPT1	Tumor Protein, Translationally-Controlled 1	1.11	2.18E-04
RPL17-C18orf32	RPL17-C18orf32	1.00	1.90E-05

Genes with logFC > 1.

Filtered by padj < 0.01.

Genes in bold were selected for qRT-PCR validation.

**Table 2:** Top 25 expressed genes in cartilage compared to outer zone meniscus tissue.

Gene symbol	Gene Name	LogFC	padj
Extracellular Matrix			
COL2A1	Collagen Type II Alpha 1 Chain	4.69	2.18E-07
**CILP2**	**Cartilage Intermediate Layer Protein 2**	**2.47**	**5.69E-08**
SPARC	Secreted Protein Acidic and Cysteine Rich	2.46	8.75E-08
ACAN	Aggrecan	2.18	4.27E-04
COL3A1	Collagen Type III Alpha 1 Chain	1.87	7.30E-06
FMOD	Fibromodulin	1.81	2.70E-05
BGN	Biglycan	1.79	3.23E-08
COMP	Cartilage Oligomeric Matrix Protein	1.61	1.32E-03
PRELP	Proline and Arginine Rich End Leucine Rich Repeat Protein	1.53	3.63E-03
Protein synthesis			
EEF2	Eukaryotic Translation Elongation Factor 2	1.57	4.87E-14
RPS27A	Ribosomal Protein S27A	1.27	1.27E-06
RPL10	Ribosomal Protein L10	1.23	1.98E-05
RPL5	Ribosomal Protein L5	1.22	2.03E-06
RPL23	Ribosomal Protein L23	1.20	1.24E-06
RPS20	Ribosomal Protein S20	1.19	2.01E-05
EEF1G	Eukaryotic Translation Elongation Factor 1 Gamma	1.14	2.12E-05
RPS3	Ribosomal Protein S3	1.12	2.13E-05
RPL4	Ribosomal Protein L4	1.06	1.20E-06
RPL6	Ribosomal Protein L6	1.01	2.02E-06
RPS8	Ribosomal Protein S8	1.01	3.52E-04
RPL8	Ribosomal Protein L8	1.01	3.75E-05
Signal Transduction			
C2orf40	ECRG4 Augurin Precursor	2.36	8.51E-03
LCN2	Lipocalin 2	1.72	1.22E-03
Transcriptional Regulation			
**AEBP1**	**AE Binding Protein 1**	**1.84**	**5.81E-03**
TPT1	Tumor Protein, Translationally-Controlled 1	1.68	3.91E-09

Genes with logFC > 1.

Filtered by padj < 0.01.

Genes in bold were selected for qRT-PCR validation.

On the other hand, the top 25 DEGs in inner zone meniscus tissue compared to cartilage showed differences in genes associated with the following: ECM, including glycoprotein *LUM*, *COL1A2*, *ECM1*, which is a matrix protein that regulates chondrogenesis and endochondral ossification^30^, matrix metalloproteinase (MMP) inhibitor *TIMP3*, and the mineralization inhibitor *MGP*; cell membrane proteins, including *KCNA6* that encodes a voltage-gated potassium channel; actin cytoskeleton (*ACTB, CAPG,* and *EZR*); mitochondrial function (*CYP1B1, CYTB*); signal transduction, including *MT2A*, which is a free radical scavenger; transcriptional regulators, including *PRRX1*, *CEBPD*, and *TRPS1*, and *HNRNPA2B1*, which encodes an RNA binding protein; enzymes, including genes involved in free radical scavenging (*SOD3*) and metabolism (*AKR1B1* and *ACSL1*); and secreted factor transglutaminase *F13A1*, which is involved in the coagulation cascade and knockout of this gene protects against collagen-induced arthritis in mice^31^ (Table 3). Of the 25 most expressed genes, the genes with greatest logFC in inner zone tissue relative to cartilage were ECM genes *LUM* (logFC = 5.34) and *MGP* (logFC = 2.81) and secreted protein *F13A1* (logFC = 2.85). The top 25 expressed genes in outer zone tissue compared to cartilage largely fell into similar categories as the inner zone, as well as including the growth factor regulator *LTBP2* (Table 4)*.* Of the top 25 most expressed genes in outer zone tissue, the genes with greatest logFC relative to cartilage were ECM genes *LUM* (logFC 6.25) and *COL1A2* (logFC 3.11) and the enzyme *MMP13* (logFC 3.00). Eleven of the top 25 expressed genes were upregulated in both inner and outer zone meniscus relative to cartilage (*CAPG, ACTB, LUM, COL1A2, ECM1, ACSL1, SOD3, CYTB, CYP1B1, PRRX1, HNRNPA2B1*).

**Table 3:** Top 25 expressed genes in inner zone meniscus tissue compared to cartilage.

Gene symbol	Gene name	LogFC	Padj
Cell Membrane Protein			
KCNA6	Potassium Voltage-Gated Channel Subfamily A Member 6	1.51	9.53E-04
SLA-11	Major Histocompatibility Complex, Class I, F	1.17	1.59E-07
PLXDC2	Plexin Domain Containing 2	1.06	1.82E-03
Cytoskeleton			
EZR	Ezrin	1.75	5.57E-30
CAPG	Capping Actin Protein, Gelsolin Like	1.14	4.08E-05
ACTB	Actin Beta	1.04	1.50E-03
Enzyme			
AKR1B1	Aldo–Keto Reductase Family 1 Member B	1.99	6.36E-09
RTN4	Reticulon 4	1.28	1.98E-07
ACSL1	Acyl-CoA Synthetase Long Chain Family Member 1	1.19	2.49E-04
SOD3	Superoxide Dismutase 3	1.10	2.46E-03
Extracellular Matrix			
**LUM**	**Lumican**	**5.34**	**1.04E-12**
MGP	Matrix Gla Protein	2.81	3.67E-03
COL1A2	Collagen Type I Alpha 2 Chain	2.20	7.65E-07
TIMP3	TIMP Metallopeptidase Inhibitor 3	1.46	1.55E-03
ECM1	Extracellular Matrix Protein 1	1.22	3.44E-03
Mitochondrial protein			
CYP1B1	Cytochrome P450 Family 1 Subfamily B Member 1	2.55	7.97E-16
CYTB	Mitochondrially Encoded Cytochrome B	1.26	2.09E-07
Secreted Protein			
F13A1	Coagulation Factor XIII A Chai	2.85	4.04E-04
Signal Transduction			
SEMA3D	Semaphorin 3D	2.62	1.88E-06
MT2A	Metallothionein 2A	1.62	7.75E-05
TNFRSF1B	TNF Receptor Superfamily Member 1B	1.00	1.12E-08
Transcriptional Regulation			
**PRRX1**	**Paired Related Homeobox 1**	**2.16**	**2.55E-26**
HNRNPA2B1	Heterogeneous Nuclear Ribonucleoprotein A2/B1	1.29	1.01E-17
CEBPD	CCAAT Enhancer Binding Protein Delta	1.14	1.04E-05
TRPS1	Transcriptional Repressor GATA Binding 1	1.03	9.87E-05

Genes with logFC > 1.

Filtered by padj < 0.01.

Genes in bold were selected for qRT-PCR validation.

**Table 4:** Top 25 expressed genes in outer zone meniscus tissue compared to cartilage.

Gene symbol	Gene name	LogFC	Padj
Cell Membrane Protein			
ANTXR1	ANTXR Cell Adhesion Molecule 1	2.07	2.59E-05
Cytoskeleton			
CRYAB	Crystallin Alpha B	2.71	5.04E-06
ACTB	Actin Beta	1.82	3.21E-09
CAPG	Capping Actin Protein, Gelsolin Like	1.81	5.70E-12
FLNA	Filamin A	1.44	3.18E-04
ACTG1	Actin Gamma 1	1.28	4.27E-11
CTNNB1	Catenin Beta 1	1.14	8.46E-06
CFL1	CFL1	1.00	6.97E-08
Enzyme			
MMP13	Matrix Metallopeptidase 13	3.00	5.81E-03
MMP3	Matrix Metallopeptidase 3	1.61	4.44E-04
ACSL1	Acyl-CoA Synthetase Long Chain Family Member 1	1.16	2.59E-04
SOD3	Superoxide Dismutase 3	1.11	1.65E-03
Extracellular Matrix			
**LUM**	**Lumican**	**6.25**	**1.95E-17**
COL1A2	Collagen Type I Alpha 2 Chain	3.11	3.22E-13
CRISPLD2	Cysteine Rich Secretory Protein LCCL Domain Containing 2	2.34	1.57E-04
ECM1	Extracellular Matrix Protein 1	1.26	1.65E-03
Growth Factor or Regulator			
LTBP2	Latent Transforming Growth Factor Beta Binding Protein 2	1.80	7.73E-04
Mitochondrial Protein			
CYP1B1	Cytochrome P450 Family 1 Subfamily B Member 1	2.31	2.27E-13
CYTB	Mitochondrially Encoded Cytochrome B	1.27	8.76E-08
Secreted Protein			
SAA2	Serum amyloid A protein	1.43	2.57E-03
Signal Transduction			
ANXA2	Annexin A2	1.07	1.03E-06
Transcriptional Regulation			
**PRRX1**	**Paired Related Homeobox 1**	**2.65**	**1.11E-39**
CCDC3	Coiled-Coil Domain Containing 3	1.90	1.82E-04
HSPA8	Heat Shock Protein Family A (Hsp70) Member 8	1.40	2.56E-08
HNRNPA2B1	Heterogeneous Nuclear Ribonucleoprotein A2/B1	1.31	2.44E-18

Genes with logFC > 1.

Filtered by padj < 0.01.

Genes in bold were selected for qRT-PCR validation.

**Fig. 2:**
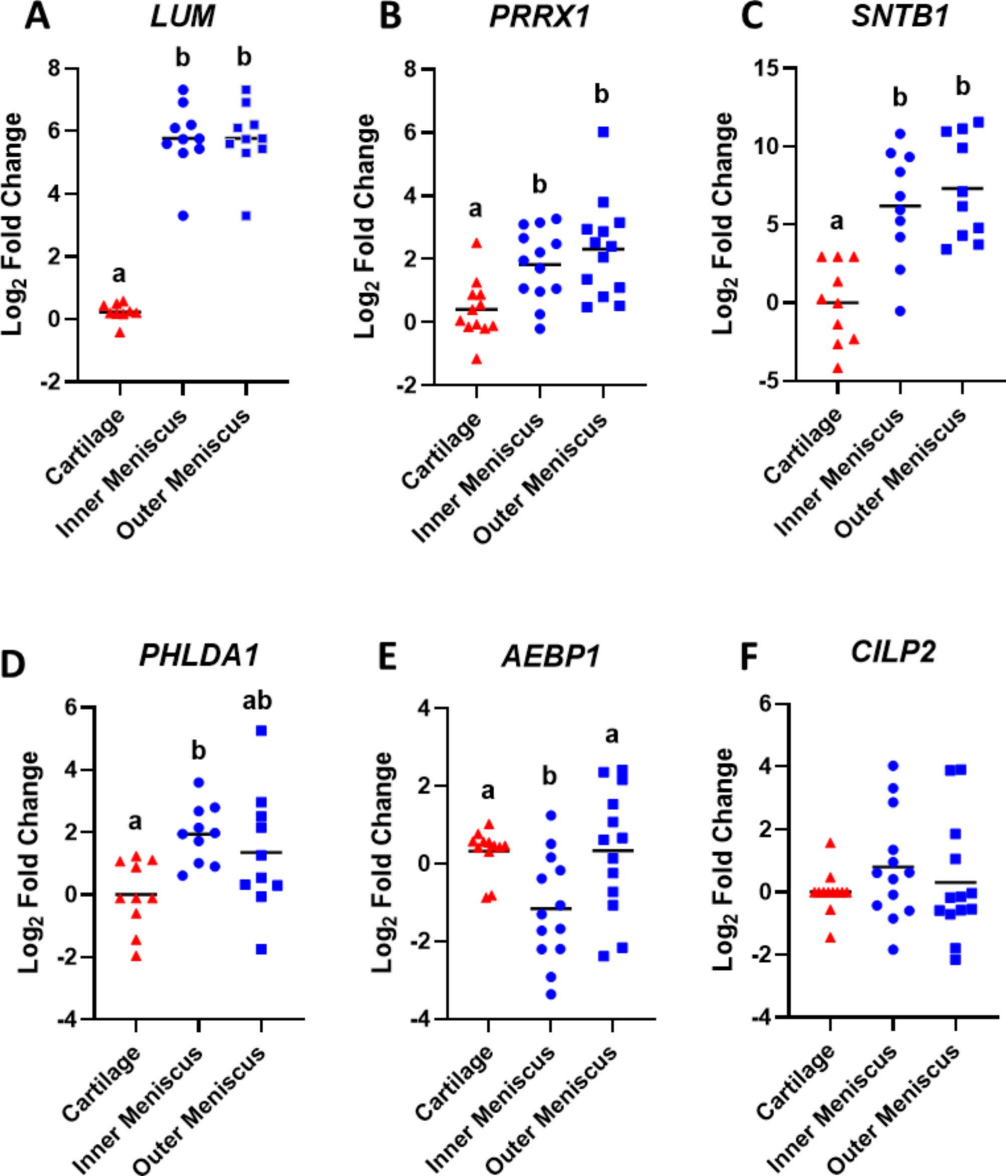
Quantitative RT-PCR for (**A**) *LUM*, (**B**) *PRRX1*, (**C**) *SNTB1*, (**D**) *PHLDA1*, (**E**) *AEBP1*, and (**F**) *CILP2* expression in cartilage and inner and outer zone meniscus tissue (N ≥ 10/group). Results are presented as log-twofold change relative to cartilage. The line indicates the mean value for each group. Groups not sharing a letter are significantly different (p < 0.05).

Several genes were selected for quantitative RT-PCR (qRT-PCR) validation of tissue specific gene expression based on logFC and significant padj. *LUM* (Fig. 2A, p < 0.001), *PRRX1* (Fig. 2B, p < 0.05), and *SNTB1* (Fig. 2C, p < 0.0001) were significantly higher in both inner and outer zone meniscus tissue compared to cartilage. Expression of *PHLDA1* was significantly higher in inner zone meniscus than cartilage (Fig. 2D, p < 0.05)*.* Interestingly, *AEBP1* was decreased in inner zone meniscus compared to both cartilage and outer zone meniscus (Fig. 2E, p < 0.05). There were no detectable differences in the expression of *CILP2* between the cartilage and meniscus tissues (Fig. 2F).

To better understand zonal differences, DEG analysis was performed on inner and outer zone meniscus, identifying a total of 205 DEGs (Supplemental Fig. 1 and Supplemental Dataset 3). The majority of these differentially expressed genes were upregulated in the outer zone tissue (190 genes) compared to the inner zone (15 genes). The most highly expressed genes in the outer zone meniscus included genes associated with the following: signal transduction, including genes related to the vasculature (*PLVAP, VWF, PECAM1*), the retinoic acid binding protein *CRABP1*, glycoproteins (*GPNMB* and *THY1*), and the glycan-binding protein *LGALS1*; cytoskeleton, including genes involved in actin regulation (*FSCN1, PLEKHO1, MYO1B,* and *LIMA1*); ECM components, such as collagens (*COL1A1*, *COL4A2*, and *COL18A1*) and glycoprotein *FBLN2*; growth factor *CCN2*, which is commonly secreted by vascular endothelial cells; and enzymes, including the matrix degrading enzyme *MMP2* (Table 5). When analyzing the top DEGs in inner zone meniscus compared to outer zone, genes associated with the following were identified: signal transduction, including genes involved in TGF-β (*CHRDL2*) and Wnt (*RSPO3*) signaling; the Ca^2^^+^ binding protein *CALR3*; ECM protein *CILP*; enzymes, including genes related to matrix degradation (*MMP16*) and metabolism (*ATP6V1B1, LNPEP,* and *FUT8*); and transcriptional regulator *OVOL1* (Table 6). Of the most highly expressed genes, the largest DEGs were *CD93* (logFC = 10.72), *PLVAP* (logFC = 9.79), and *VWF* (logFC = 9.41) in the outer zone and *CHRDL2* (logFC = 5.31) and *RSPO3* (logFC = 4.55) for the inner zone meniscus.

**Table 5:** Top 25 expressed genes in outer zone meniscus tissue compared to inner zone meniscus tissue.

Gene symbol	Gene name	LogFC	padj
Cytoskeleton			
CD93	CD93 Molecule	10.72	2.76E-05
FSCN1	Fascin Actin-Bundling Protein 1	2.61	3.54E-04
PLEKHO1	Pleckstrin Homology Domain Containing O1	2.53	2.22E-03
MYO1B	Myosin IB	2.07	7.09E-03
TAGLN2	Transgelin 2	1.87	2.61E-04
LIMA1	LIM domain and actin binding 1	1.51	1.91E-03
Extracellular Matrix			
COL1A1	Collagen Type I Alpha 1 Chain	6.30	1.11E-09
COL4A2	Collagen Type IV Alpha 1 Chain	4.43	8.10E-04
POSTN	Periostin	3.82	5.07E-03
FBLN2	Fibulin 2	2.54	4.17E-04
COL18A1	Collagen Type XVIII Alpha 1 Chain	2.30	4.09E-03
Enzyme			
MMP2	Matrix Metallopeptidase 2	2.89	4.24E-04
ANPEP	Alanyl Aminopeptidase	2.74	1.06E-03
UBE2L6	Ubiquitin Conjugating Enzyme E2 L6	1.40	8.37E-03
Growth factor			
CNN2	Calponin 2	1.03	5.70E-03
Signal transduction			
PLVAP	Plasmalemma Vesicle Associated Protein	9.79	2.20E-05
VWF	Von Willebrand Factor	9.41	4.86E-05
PECAM1	Platelet And Endothelial Cell Adhesion Molecule 1	4.72	2.85E-04
CRABP1	Cellular Retinoic Acid Binding Protein 1	4.63	3.59E-06
GPNMB	Glycoprotein Nmb	4.25	9.20E-04
LGALS1	Galectin 1	2.44	3.80E-03
TMEM119	Transmembrane Protein 119	2.35	3.93E-03
THY1	Thy-1 Cell Surface Antigen	1.58	4.02E-03
Transcriptional regulation			
NFATC4	Nuclear factor of activated T-cells 4	1.26	9.12E-03
Unknown			
PALM2	Paralemmin 2	1.19	8.54E-03

Genes with logFC > 1.

Filtered by padj < 0.01.

**Table 6:** Most highly expressed genes in inner zone meniscus tissue compared to outer zone meniscus tissue.

Gene symbol	Gene name	LogFC	padj
Ca2 + Binding Protein			
CALR3	Calreticulin 3	1.34	9.45E-03
Cytoskeleton			
SEMA3E	Semaphorin 3E	2.67	6.52E-04
Extracellular Matrix			
CILP	Cartilage Intermediate Layer Protein 2	2.35	1.68E-03
Enzyme			
ATP6V1B1	ATPase H + Transporting V1 Subunit B1	2.97	8.10E-03
MMP16	Matrix Metallopeptidase 16	1.63	1.46E-03
LNPEP	Leucyl And Cystinyl Aminopeptidase	1.20	1.15E-04
FUT8	Fucosyltransferase 8	1.01	7.77E-03
Signal transduction			
CHRDL2	Chordin Like 2	5.31	9.09E-03
RSPO3	R-Spondin 3	4.55	9.30E-03
FIBIN	Fin Bud Initiation Factor Homolog	1.84	6.02E-03
NXPH4	Neurexophilin 4	1.66	1.45E-04
STC2	Stanniocalcin 2	1.44	6.51E-03
ENKUR	Enkurin	1.04	9.77E-03
Transcriptional regulation			
OVOL1	Ovo Like Transcriptional Repressor 1	2.53	3.15E-03
Unknown			
CRISPLD1	Cysteine Rich Secretory Protein LCCL Domain Containing 1	2.11	4.09E-03

Genes with logFC > 1.

Filtered by padj < 0.01.

**Fig. 3:**
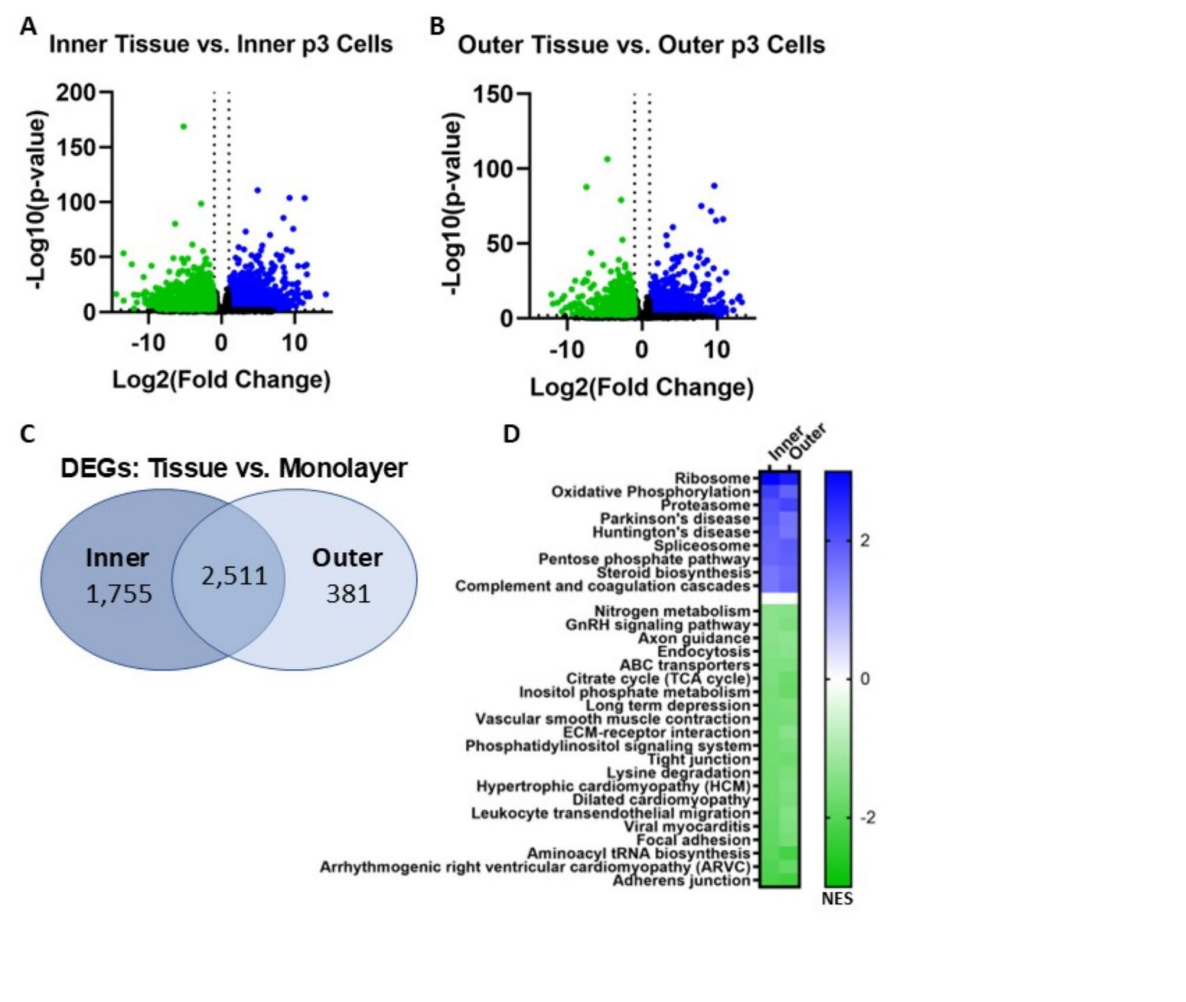
Comparison of Tissue vs. Passage 3 (p3) Monolayer Meniscus Cells using Differentially Expressed Gene Analysis and Gene Set Enrichment Analysis (GSEA). Volcano plots show genes significantly differentially expressed between (**A**) inner zone tissue compared to p3 inner zone meniscus cells and (**B**) outer zone meniscus tissue versus p3 outer zone meniscus cells. Each data point is an individual gene. Dashed lines indicate cutoffs for significant genes (FDR adjusted p-value < 0.01 and a |base-2 log fold-change|> 1). Color denotes if the gene was significantly up-regulated (blue) or down-regulated (green) in meniscus tissue relative to P3 cells, or not significantly different (black). (**C**) Number of significantly differentially expressed genes (DEGs) exclusive to inner zone tissue compared to inner zone p3 cells, outer zone tissue compared to outer zone p3 cells, or found in both zones compared to their respective p3 cells. (**D**) GSEA of inner meniscus tissue versus inner p3 meniscus cells and outer meniscus tissue versus outer p3 meniscus cells. Normalized enrichment scores (NES) of pathways significantly regulated by monolayer culturing in both inner and outer zone samples are shown. A NES > 0 (blue) identifies upregulated pathways and NES < 0 (green) identifies downregulated pathways.

The most abundant differences in gene expression were identified when comparing meniscus tissue to passaged meniscus cells. The comparison of inner zone meniscus tissue to passaged inner zone cells yielded 4,266 significant DEGs with 2,122 up-regulated and 2,144 down-regulated in tissue compared to passaged inner zone cells (Fig. 3A and Supplemental Dataset 4). A total of 2,892 significant DEGs were identified between outer zone tissue and passaged outer zone cells (1,573 up-regulated; 1,319 down-regulated) (Fig. 3B and Supplemental Dataset 5). There was substantial overlap in DEGs, with 2,511 of the transcripts identified being shared between both comparisons (Fig. 3C). However, 1,755 genes were unique in the inner zone comparison and 381 genes were unique in the outer zone tissue to passaged cell comparison.

To better identify biological processes associated with gene expression differences between meniscus tissue and passaged cells, Gene Set Enrichment Analysis (GSEA) using the KEGG geneset database was performed. Numerous overlapping KEGG pathways (30 pathways) with an FDR < 0.25 were found between inner and outer zone tissue comparisons to passaged cells from the corresponding zone (Fig. 3D). Nine of these pathways were upregulated in meniscus tissue relative to monolayer cultured cells, while 21 pathways were upregulated in monolayer cells.

When comparing the top 25 genes expressed in passaged inner zone cells compared to inner zone tissue (Table 7) and passaged outer zone cells compared to outer zone tissue (Table 8), genes associated predominantly with the cytoskeleton and ECM were upregulated. In particular, upregulation of genes in the actin, myosin, and tropomyosin families were evident. In the top 25 most expressed genes, the most highly DEGs in passaged inner zone cells relative to tissue were *COL1A1* (logFC = 13.40), *ACTA2* (logFC = 12.27), and *TAGLN* (logFC = 6.88). In the passaged outer zone cells relative to tissue, the most highly DEGs were *SFRP2* (logFC = 10.38), *ACTA2* (logFC = 7.95), and *IGFBP2* (logFC = 7.89). Twenty-two of the 25 genes were upregulated in passaged cells from both zones compared to tissue from the corresponding zone.

**Table 7:** Top 25 expressed genes in inner zone P3 meniscus cells compared to inner zone tissue.

Gene symbol	Gene name	LogFC	padj
Extracellular Matrix			
COL1A1	Collagen Type I Alpha 1 Chain	13.40	4.58E-54
COL1A2	Collagen Type I Alpha 2 Chain	5.34	2.13E-38
FBN1	Fibrillin 1	4.63	8.31E-37
COL5A1	Collagen Type V Alpha 1 Chain	3.87	5.65E-16
COL5A2	Collagen Type V Alpha 2 Chain	3.37	7.73E-28
COL3A1	Collagen Type III Alpha 1 Chain	3.08	7.78E-15
FLNA	Filamin A	3.04	6.53E-16
COL11A1	Collagen Type XI Alpha 1 Chain	2.83	1.26E-07
THBS1	Thrombospondin 1	2.56	1.33E-07
TNC	Tenascin C	2.11	1.30E-04
SPARC	Secreted Protein Acidic and Cysteine Rich	2.06	5.23E-06
COL6A1	Collagen Type VI Alpha 1 Chain	1.79	4.38E-06
Cytoskeleton			
**ACTA2**	**Actin Alpha 2, Smooth Muscle**	**12.27**	**3.67E-44**
**TAGLN**	**Transgelin**	**6.88**	**2.34E-21**
TPM1	Tropomyosin 1	5.17	1.80E-169
CALD1	Caldesmon 1	5.11	3.09E-45
MYH9	Myosin Heavy Chain 9	3.26	1.04E-11
TPM4	Tropomyosin 4	2.59	1.46E-35
ACTB	Actin Beta	1.79	2.16E-09
ACTG1	Actin Gamma 1	1.43	3.64E-14
VIM	Vimentin	2.50	1.94E-19
LMNA	Lamin A/C	1.65	5.87E-15
Growth factor			
IGFBP5	Insulin Like Growth Factor Binding Protein 5	1.87	2.31E-05
Transcription Factor			
AEBP1	AE Binding Protein 1	3.92	2.57E-10
**FSTL1**	**Follistatin Like 1**	**2.19**	**1.93E-20**

Genes with logFC > 1.

Filtered by padj < 0.01.

Genes in bold were selected for qRT-PCR validation.

**Table 8:** Top 25 expressed genes in outer zone P3 meniscus cells compared to outer zone tissue.

Gene symbol	Gene name	LogFC	padj
Cytoskeleton			
**ACTA2**	**Actin Alpha 2, Smooth Muscle**	**7.95**	**1.33E-15**
**TAGLN**	**Transgelin**	**5.93**	**1.00E-12**
TPM1	Tropomyosin 1	4.60	3.90E-107
CALD1	Caldesmon 1	4.15	8.46E-24
MYH9	Myosin Heavy Chain 9	2.51	6.75E-06
TPM4	Tropomyosin 4	2.19	2.51E-20
ACTB	Actin Beta	1.31	1.97E-04
VIM	Vimentin	1.86	7.22E-09
LMNA	Lamin A/C	1.33	5.05E-08
Extracellular Matrix			
COL1A1	Collagen Type I Alpha 1 Chain	6.66	4.19E-11
COL1A2	Collagen Type I Alpha 2 Chain	4.09	4.49E-18
COL4A2	Collagen Type IV Alpha 2 Chain	3.70	2.10E-03
THBS1	Thrombospondin 1	3.68	2.00E-11
FBN1	Fibrillin 1	3.66	2.21E-18
COL5A1	Collagen Type V Alpha 1 Chain	3.64	3.03E-11
COL3A1	Collagen Type III Alpha 1 Chain	3.59	1.32E-15
COL5A2	Collagen Type V Alpha 2 Chain	2.85	6.46E-16
FLNA	Filamin A	2.46	1.58E-08
COL11A1	Collagen Type XI Alpha 1 Chain	2.44	9.41E-05
SPARC	Secreted Protein Acidic and Cysteine Rich	2.40	3.50E-06
COL4A1	Collagen Type VI Alpha 1 Chain	1.29	5.61E-03
Growth factor			
**SFRP2**	**Secreted Frizzled Related Protein 2**	**10.38**	**1.08E-15**
IGFBP2	Insulin Like Growth Factor Binding Protein 2	7.89	2.13E-24
IGFBP5	Insulin Like Growth Factor Binding Protein 5	2.80	1.92E-08
Transcription Factor			
AEBP1	AE Binding Protein 1	2.01	7.43E-03

Genes with logFC > 1.

Filtered by padj < 0.01.

Genes in bold were selected for qRT-PCR validation.

Comparison of the top 25 genes expressed in inner zone meniscus tissue compared to passaged inner zone meniscus cells (Table 9) and outer zone meniscus tissue compared to passaged outer zone meniscus cells (Table 10) identified upregulated genes associated with the following: ECM, including matrix proteins *ACAN, COMP, DCN*, and *FN1*; membrane glycoprotein *MFGE8*; mitochondria, including *SOD2* and *COX3*, which are involved in oxidative phosphorylation; growth factors and regulators, including *TPT1*, which is a regulator of cell growth and proliferation; iron trafficking (*LCN2*) and storage (*FTL* and *FTH1*); ribosomal proteins, such as *RPS2*; signal transduction, including *SAA3,* which enables Toll-like receptor binding, and *CLU*, which has anti-apoptotic, anti-inflammatory and antioxidant properties in cartilage^32^; enzymes, including those involved in matrix degradation (*CTSD*) and metabolism (*GAPDH*); and secreted protein *SAA1*, which plays a role in inflammation and tissue injury. Nineteen of these highly expressed genes in meniscus inner and outer zone tissue were shared. The top-most DEGs in inner zone tissue compared to passaged cells were *COL2A1* (logFC = 8.63), *SAA3* (logFC = 8.47), and *SAA1* (logFC = 8.05). In outer zone tissue, *SAA3* (logFC = 9.65) and *SAA1* (logFC = 9.31) were also among the most DEGs, as well as *LCN2* (logFC = 7.79).

**Table 9:** Top 25 expressed genes in inner zone tissue compared to inner zone P3 meniscus cells.

Gene symbol	Gene name	LogFC	padj
Cell Membrane Protein			
MFGE8	Milk Fat Globule-EGF Factor 8 Protein	3.32	1.58E-16
Extracellular Matrix			
COL2A1	Collagen 2A1	8.63	1.49E-23
HAPLN1	Hyaluronan and Proteoglycan Link Protein 1	6.34	4.01E-28
MGP	Matrix G1a	6.03	4.43E-12
SPP1	Secreted Phosphoprotein 1	5.35	6.51E-38
ACAN	Aggrecan	4.54	6.18E-15
TIMP3	Tissue inhibitor of metalloproteinase 3	4.46	3.24E-27
COMP	Cartilage oligomeric matrix protein	4.35	1.19E-20
DCN	Decorin	2.88	1.74E-21
PRELP	Proline and arginine rich end leucine rich repeat protein	2.24	6.85E-06
FN1	Fibronectin 1	1.76	7.21E-04
FMOD	Fibromodulin	1.67	7.26E-05
Enzyme			
CTSD	Cathepsin D	2.61	1.97E-15
GAPDH	Glyceraldehyde-3-Phosphate Dehydrogenase	2.02	2.09E-20
Growth Factors and Regulators			
TPT1	Tumor protein, Translationally-Controlled 1	1.84	3.01E-11
Iron Homeostasis			
LCN2	Lipocalin 2	6.88	4.46E-45
FTL	Ferritin light chain	2.76	4.88E-17
FTH1	Ferritin heavy polypeptide 1	2.73	1.29E-27
Mitochondrial Protein			
SOD2	Superoxide dismutase 2	5.59	2.83E-61
COX3	Cytochrome C Oxidase subunit III	1.47	7.80E-17
Ribosomal Protein			
RPS2	Ribosomal protein S2	1.76	6.02E-10
RPLP0	Ribosomal protein lateral stalk subunit P0	1.15	4.00E-06
Secreted Protein			
SAA1	Serum amyloid A protein 1	8.05	4.32E-04
Signal Transduction			
SAA3	Serum amyloid A protein	8.47	2.65E-86
CLU	Clusterin	5.33	6.74E-45

Genes with logFC > 1.

Filtered by padj < 0.01.

**Table 10:** Top 25 expressed genes in outer zone tissue compared to outer zone P3 meniscus cells.

Gene symbol	Gene name	LogFC	padj
Cell Membrane Protein			
MFGE8	Milk Fat Globule-EGF Factor 8 Protein	2.44	1.91E-07
Extracellular Matrix			
SPP1	Secreted Phosphoprotein 1	6.46	1.23E-43
MGP	Matrix G1a	6.16	6.58E-10
ACAN	Aggrecan	4.69	1.61E-12
COMP	Cartilage oligomeric matrix protein	2.86	1.57E-07
DCN	Decorin	2.64	2.22E-14
CCDC80	Coiled-coil domain containing 80	2.02	1.11E-03
FN1	Fibronectin 1	1.97	1.10E-03
Enzyme			
CTSD	Cathepsin D	2.29	1.41E-09
HTRA1	Serine protease HTRA1	1.95	4.67E-06
GAPDH	Glyceraldehyde-3-Phosphate Dehydrogenase	1.86	7.47E-14
CYP1B1	CYP450 Family 1 Subfamily B Member 1	1.16	1.80E-03
Growth Factors and Regulators			
NBL1	DAN Family BMP antagonist	3.96	7.91E-34
TPT1	Tumor protein, Translationally-Controlled 1	1.12	6.62E-04
Iron Homeostasis			
LCN2	Lipocalin 2	7.79	1.24E-45
FTH1	Ferritin heavy polypeptide 1	2.48	4.07E-18
FTL	Ferritin light chain	1.78	3.56E-06
Mitochondrial Protein			
SOD2	Superoxide dismutase 2	5.19	5.06E-42
SOD3	Superoxide dismutase 3	3.05	2.17E-16
ATP6	ATP synthase 6	2.19	4.07E-13
COX3	Cytochrome C oxidase subunit III	1.24	1.17E-09
Ribosomal Protein			
RPS2	Ribosomal protein S2	1.23	2.46E-04
Secreted Protein			
SAA1	Serum amyloid A protein 1	9.31	4.24E-04
Signal Transduction			
SAA3	Serum amyloid A protein	9.65	3.07E-89
CLU	Clusterin	4.90	4.42E-30

Genes with logFC > 1.

Filtered by padj < 0.01.

**Fig. 4:**
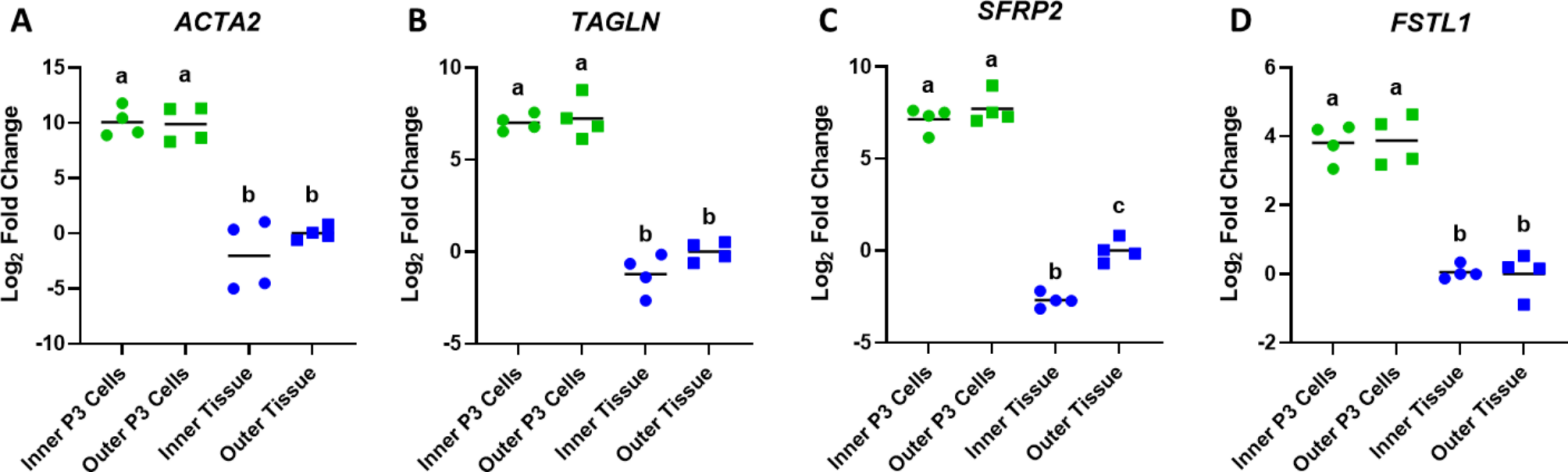
qRT-PCR for (**A**) *ACTA2*, (**B**) *TAGLN*, (**C**) *SFRP2*, and (**D**) *FSTL1* expression in passage 3 (P3) inner and outer zone meniscus cells and inner and outer zone meniscus tissue (n = 4/group). Results are presented as log-twofold change relative to the meniscus outer zone tissue. The line indicates the mean value for each group. Groups not sharing a letter are significantly different (p < 0.001).

Quantitative RT-PCR was used to validate expression of *ACTA2* (Fig. 4A), *TAGLN* (Fig. 4B), *SFRP2* (Fig. 4C), and *FSTL1* (Fig. 4D) in passaged cells and meniscus tissue obtained from both the inner and outer zones. Expression of each of these genes was upregulated in both the inner and outer zone passaged cells compared to inner and outer zone tissue (p < 0.0001). There were no detectable differences in expression between passaged cells obtained from the inner and outer zone or tissue obtained from either zone except for *SFRP2,* which was downregulated in inner zone meniscus tissue compared to outer zone meniscus tissue (p < 0.001).

## Discussion

Despite the common comparison of inner zone meniscus cells to chondrocytes and outer zone meniscus cells to fibroblasts^7,8,11^, our findings show that inner and outer zone meniscus cells have a distinct transcriptomic profile and are more similar to each other than to either articular cartilage or passaged meniscus cells. When comparing by PCA, it is noted that inner meniscus tissue clusters slightly closer to cartilage and outer zone meniscus tissue clusters closer to monolayer cells. However, differences between meniscus tissue and articular cartilage or monolayer cells are much greater than differences between the zones of the meniscus. Similarly, DEG analysis shows that inner and outer zone meniscus tissues are more closely related to each other than to articular cartilage or monolayer cultured cells. More than 3,100 DEGs were identified between meniscus tissue and cartilage compared to 205 between inner and outer zone meniscus tissue. Of these DEGs between meniscus and cartilage, 1337 were shared between inner and outer meniscus, suggesting that the transcriptome of inner and outer meniscus cells differ from cartilage in a similar way. Interestingly, prior work using single cell RNA-Seq revealed new chondrocyte subtypes that appear in degenerated human meniscus tissue, suggesting that these chondrocyte populations in the meniscus tissue may be therapeutic targets^33^. When comparing meniscus tissue and passaged cells, more than 4,600 DEGs were identified. These data show that in situ meniscus cells are a distinct cell type, substantially different from chondrocytes in articular cartilage, and these cells undergo significant transcriptomic changes when passaged.

When looking at the top 25 abundantly expressed genes in cartilage compared to meniscus tissue, there were major differences in ECM-associated genes, as well as genes associated with signal transduction and transcriptional regulation. When comparing cartilage to outer zone meniscus tissue, several expected genes, including *COL2A1* and *ACAN* were identified. Cartilage experiences more compressive loads than the outer zone of the meniscus. Therefore, in cartilage, the relatively increased expression of genes that encode negatively-charged proteoglycans^34^ that can resist compressive loads, such as *ACAN, FMOD*, and *BGN,* is expected. Interestingly, this analysis also identified the upregulation of numerous ribosomal proteins in cartilage compared to outer zone tissue. While ribosomal proteins play an essential role in protein translation, their extra-ribosomal functions, including roles in tumorigenesis, immune signaling, and development^35^ may prove to be interesting targets for future studies. On the other hand, the top 25 expressed genes in meniscus compared to cartilage include previously studied genes such as *COL1A2*, as well as several other genes associated with ECM, mitochondrial proteins, signal transduction, and proteins related to transcriptional regulation^36,37^. Increased expression of genes encoding mitochondrial proteins in meniscus tissue compared to cartilage, such as *CYP1B1* and *CTYB,* may point to differences in metabolism between the tissues, while increased expression of actin family genes *CAPG*, *ACTB,* and *ACTG1* may indicate differences in cell mechanobiology, migration, and/or motility^38^.

PCR validation of several genes showed trends that largely aligned with our RNA-seq results. In particular, meniscus tissue showed significantly higher expression of *LUM, PRRX1,* and *SNTB1* compared to cartilage. Previous studies in mice homozygous for a null mutation of the small leucine-rich proteoglycan lumican showed reduced tensile strength, as well as thicker collagen fibrils and non-uniform interfibrillar spacing in other connective tissues^39^. Therefore, increased expression of *LUM* in meniscus may indicate an important role of lumican in collagen structure, function, and matrix organization. Prior work has shown upregulation of *LUM* in meniscus but not cartilage following anterior cruciate ligament transection in rats^40^ but an increase in lumican fragmentation in degenerated human cartilage and menisci^41^. *PRRX1*, a transcription factor that binds to the promoter region of TGF-β1, is associated with early limb development, joint morphogenesis^42,43^, regulation of proliferation and differentiation^44^, and can promote meniscus repair^45^. While not one of the most abundantly expressed genes in the inner or outer zone meniscus tissue, *SNTB1* was highly upregulated in both the inner and outer zone tissue compared to cartilage. While the specific role of *SNTB1* has not been studied in meniscus, its role in Wnt/β-catenin signaling has implications in OA development^46–48^. Interestingly, expression of *PHLDA1* and *AEBP1* was specific to meniscus tissue zone with *PHLDA1* expression increased in inner zone meniscus tissue compared to cartilage and *AEBP1* expression decreased in inner zone meniscus tissue compared to cartilage and outer zone meniscus. *PHLDA1* encodes a protein involved in apoptosis and upregulation of this gene is associated with reduced cell growth and cell death^49,50^, while *AEBP1* encodes for secreted aortic carboxypeptidase-like protein (ACLP), which plays a role in collagen polymerization^27^. Variants in AEBP1 cause ultrastructural changes in collagen organization and have been identified in patients with Ehlers-Danlos^27^, which clinically results in poor wound healing, joint hypermobility, and osteoporosis^51^. The differential expression of these two genes may have interesting implications in the collagen structure and differential healing abilities between the inner and outer zones. Overall, these robustly and differentially expressed genes provide interesting targets for further research in distinguishing between cartilage and meniscus tissue.

Our findings illustrate the global transcriptomic differences between inner and outer zone meniscus tissue. The larger number of upregulated genes in the outer zone is likely due to the presence of vasculature and vascular-associated cells in the outer zone, which are not found in the inner avascular zone^52^. Furthermore, differences in gene expression profiles between the zones may also be driven by the structural adaptations of the tissue, which is built to withstand the different magnitudes of compressive and tensile strains^53^ in vivo. The expression and arrangement of collagen fibers in the meniscus tissue is critically important for resisting the circumferential hoop stresses experienced by the tissue. The outer zone expressed higher levels of several collagens than the inner zone, including *COL1A1, COL4A2*, and *COL18A1. CILP* was the only ECM component that was upregulated in the inner zone compared to outer zone but the function of CILP in the meniscus tissue is currently unclear. Interestingly, CILP protein levels are decreased in end-stage OA menisci^54^; however, this study did not compare inner and outer zone protein concentrations. While PCA largely showed separation of inner and outer zone meniscus tissue, there was one outer zone sample that clustered with the inner zone tissue samples. There is no physical delineation between the inner and outer zones of the meniscus. Indeed, some studies separate the outer third from the inner two-thirds^10,17,18^, while others separate at the midline of the tissue^55^ or into thirds^9,11^. Explants for RNA-seq were harvested from the inner two-thirds and outer one-third of the meniscus. Interestingly, the two meniscus tissue samples that overlapped in the PCA were taken closer to the anterior and posterior horn, while the other samples were taken closer to the mid-body of the meniscus. It may be that the inner/outer delineation becomes less relevant approaching the horn region and/or that the cells of the horn region are a third distinct phenotype^56^. Indeed, it is likely that meniscus cells vary in all three dimensions, as differences between cells of the superficial and deep regions have also been reported^11,57^. To diminish the confounding effects of potential tissue overlap, samples obtained for qRT-PCR validation were harvested from only the inner one-third and outer one-third of the meniscus tissue, which may explain some of the differences detected between the RNA-seq findings and qRT-PCR. This further separation allowed the detection of significantly lower expression of *AEBP1* and *SFRP2* in the inner zone tissue as compared to the outer zone tissue. *SFRP2* is a marker for bone marrow-derived stem cells and skeletal stem cell multipotency^58^. Given the vascular distribution in meniscus tissue, it is possible that multipotent stem cells may be localized more to the outer zone than the inner zone. While our study has identified overall transcriptomic differences between zones, further work must be performed to identify distinct cell populations between zones of the meniscus. Single-cell sequencing techniques have been utilized to elucidate the cellular landscape of human menisci and how these change with tissue degeneration^33,59^. Leveraging single-cell sequencing analysis may help to determine whether these zonal transcriptomic differences represent phenotypic changes of a single predominant meniscal cell type or changing proportions of multiple cell types.

In this study, we also revealed profound global transcriptomic changes that occur due to passaging of meniscus cells. There are more DEGs and larger fold-change differences between meniscus tissue and passaged cells than any other pairwise comparison. The passaged cells were not donor-matched to the tissue samples, which may have decreased the specificity of RNA-seq to identify changes specific to monolayer passaging. However, the qPCR validation of select genes was performed in a separate cohort of donor samples for both tissue and isolated cells, supporting the generalizability of the measured DEGs. The majority of DEGs and KEGG pathways were shared between inner and outer zone comparisons, suggesting that passaging results in cells that are transcriptionally similar regardless of zone of origin and that the process of dedifferentiation may be similar as well. This is also evidenced by the tight clustering of inner and outer zone cells on the PCA plots. When analyzing GSEA data, metabolic pathways were well-represented in the comparison of meniscus tissue to passaged cells. Oxidative phosphorylation, pentose phosphate pathway, and steroid biosynthesis were all more highly expressed in tissue than passaged cells, while citrate cycle (TCA), nitrogen metabolism, and inositol phosphate metabolism were more highly expressed in passaged cells, suggesting a major shift in energy metabolism that occurs with cellular dedifferentiation. Passaged cells also exhibited increased expression of pathways related to cell–cell and cell–matrix interactions, including adherens junction, focal adhesion, tight junction, and ECM-receptor interaction, indicating transcriptional changes related to their physical environment. Identification of some unexpected pathways, such as arrhythmogenic right ventricular cardiomyopathy (ARVC) and viral myocarditis, were likely due to cytoskeletal changes as many of the top genes driving these associations are members of the integrin, myosin, and actin families. Given that the tissue explants were maintained in the same culture medium as the monolayer cells for three days before RNA extraction, these transcriptomic findings are not attributable to in vitro culture. However, alterations in the physical microenvironment likely predominantly drive these changes and our findings reflect the effect of removing the three-dimensional ECM-cell interactions and placing cells in a two-dimensional culture dish. While it is known that primary meniscus cells dedifferentiate in culture^8–12^, prior studies have largely focused on just a few genes based on changes observed during chondrocyte dedifferentiation. Therefore, our data provides an unbiased comprehensive picture of the global gene expression changes in both the inner and outer zone cells due to passaging and provides a broader set of meniscus dedifferentiation markers that can be utilized in meniscus biology and tissue engineering studies to recapitulate the native meniscus phenotype.

When assessing the top 25 expressed genes in monolayer passaged meniscus cells, numerous cytoskeleton-associated transcripts, particularly in the actin and tropomyosin families, were upregulated compared to meniscus tissue. Changes in expression of *ACTA2*, smooth muscle alpha-2 actin, and *TGLN,* an early marker of smooth muscle differentiation*,* together with alterations in actin polymerization and cell morphology have previously been implicated in chondrocyte dedifferentiation^60^. These changes in gene expression indicate an acquisition of contractile properties, which may have major implications for tissue engineering applications. *ACTA2* expression has been identified as an important marker in meniscus tissue healing^61^ and smooth muscle actin immunostaining is increased in the superficial zone of torn menisci but is absent in normal and OA menisci^57^. However, studies in chondrocytes have shown that excessive tissue contraction and shrinkage secondary to expression of contractile proteins may prevent integration of implanted tissues^60^. Therefore, further work is needed to understand the role of ACTA2 in meniscus tissue healing. *FSTL-1*, which was also upregulated by meniscus cell passaging, is associated with activation of NF-κB signaling pathways, an important pathway for inflammatory signaling and OA development^62,63^, and thus its upregulation in passaged cells may have implications for in vitro OA studies. Finally, expression of both *SFRP2* and *TAGLN* were upregulated in passaged cells compared to meniscus tissue. *SFRP2*, a modulator of canonical Wnt signaling, is expressed in the inner and middle regions of the embryonic mouse meniscus^64^. While the roles of these genes have not been studied in meniscus, their increased expression in passaged meniscus cells suggests these are markers of the dedifferentiated passaged meniscal cells. Transcriptomic data from this study serves as an important foundation for exploring the factors and pathways that drive dedifferentiation and provides benchmarks for restoring the native meniscus phenotype.

Future studies are necessary to address some of the limitations of this work. In this study, due to tissue availability from our abattoir, we were only able to assess female tissues. Analyzing the effect of sex on these transcriptomic findings is an important next step. Furthermore, in this study, we used a 3-day preculture period for the tissue samples prior to RNA extraction. This step was intended to standardize culture conditions experienced by the tissues and cells to allow direct comparison of the expression profiles of the cells existing in the native 3D ECM versus monolayer culture on tissue culture plastic, including nutrient and gas composition of the media and incubation temperature. The preculture period most likely resulted in transcriptomic changes relative to the in vivo state^12^. However, in this study, the preculture period allowed us to compare tissues and cells that had experienced similar culture conditions to isolate the effects of the 3D ECM and cell passaging. In addition to comparisons of freshly isolated porcine tissues, it would also be interesting to investigate differences in human meniscus and cartilage samples.

Overall, we have identified and characterized the global transcriptomic differences between cartilage, inner and outer zone meniscus tissue, and passaged meniscus cells, allowing us to reveal differences between these connective tissues and changes in phenotype due to monolayer culturing. Comparing the expression profiles of cultured meniscus cells to meniscus tissue resident cells has revealed the radical transcriptomic changes that occur during in vitro cell passaging. These results provide comprehensive, unbiased transcriptomic changes and numerous novel targets for characterizing inner and outer zone meniscus cells and tissue constructs for regenerative medicine approaches. We have validated *LUM, PRRX1,* and *SNTB1* as markers for meniscus tissue and *ACTA2*, *TAGLN*, *SFRP2*, and *FSTL1* as novel markers for meniscus cell dedifferentiation. Finally, this work provides comprehensive meniscus gene expression profiles and can serve as a resource for future studies of meniscus cell biology, regenerative medicine, and tissue engineering.

## Materials and methods

### RNA sequencing experiments

#### Tissue sample collection

Porcine stifle joints from skeletally mature 2–3 year old female pigs were obtained from a local abattoir. Explants from cartilage (N = 3) were harvested using a scalpel to remove full thickness sections of articular cartilage from the femoral surface, which were approximately 5 mm x 5 mm. Meniscus tissue explants were obtained using a 5 mm biopsy punch inserted perpendicular to the femoral surface of the meniscus. Explants were cut to approximately 2 mm in depth, preserving the femoral surface for culture. Explants were taken from the inner two-thirds and outer one-third of the medial menisci (N = 3/zone; Supplemental Fig. 2) and placed in DMEM-HG (DMEM-HG; Catalog # 11,995,073; Gibco, Carlsbad, CA) with 10X antibiotic/antimycotic (Catalog # 15,240,062; Gibco) for one hour. Explants were subsequently maintained in culture media composed of DMEM-HG, 10% fetal bovine serum (Catalog # SH30396.03; HyClone, Logan, UT), 1X non-essential amino acids (Catalog # 11,140,050; Gibco), 1X antibiotic/antimycotic, 10 mM HEPES (Cat # 15,630,080; Gibco), 40 μg/mL L-proline (SKU # H54409; Sigma-Aldrich, St. Louis, MO), and 50 μg/mL ascorbate (SKU # A8960; Sigma-Aldrich). Media was changed the day after explants were harvested and explants were cultured for an additional two days at 37 °C, 5% CO_2_ for a total of three days. Samples were rinsed in phosphate buffered saline (PBS; catalog # 10,010–023; Gibco), flash frozen in liquid nitrogen, and stored at -80 °C until RNA extraction.

#### Tissue RNA extraction

Explants were pulverized in TRIzol (Catalog # 15,596,026; Life Technologies, Carlsbad, CA) using a freezer mill (Model 6875; SPEX SamplePrep, Metuchen, NJ) under liquid nitrogen^12,21^. Samples underwent three, two-minute cycles at maximum frequency with two minutes precooling between cycles. RNA was extracted by TRIzol/chloroform separation, resuspended in lysis buffer, and column purified according to the Norgen Total RNA Purification Plus extraction kit protocol (Catalog # 48,300; Norgen Biotek Corp, Ontario, Canada).

#### Cell isolation and monolayer culture

Meniscus cells were isolated from the outer one-third or inner two-thirds of the porcine medial meniscus (N = 3 inner zone, N = 2 outer zone). Tissue was minced and processed by sequential digestion with 0.5% (w/v) pronase (SKU 53,702; EMD Millipore Corp., Temecula, CA) for one hour and 0.2% (w/v) collagenase type I (Catalog # LS004197; Worthington Biochemical Corp., Lakewood, NJ) for 16 h in complete culture media^12,21^. Cells were seeded at 5 million cells per 10 cm tissue culture plate and maintained in complete culture media. Media was replaced every 2–3 days and cells were passaged at 90% confluence (5–6 days for passage 1 and then 2–3 days for subsequent passages). Cell pellets were collected at the third passage, flash frozen in liquid nitrogen, and stored at -80 °C until RNA extraction was performed using the Norgen Total RNA Purification Plus extraction kit.

#### RNA sequencing

RNA-seq was performed by the Duke Center for Genomic and Computational Biology (Durham, NC). Reads that were 20nt or longer after trimming were mapped to the Sscrofa11.1v91 version of the pig genome and transcriptome^65^, using the STAR RNA-seq alignment tool^66^. Subsequent analysis was performed only for genes that mapped to a single genomic location. Using the HTSeq tool, gene counts were compiled, and subsequent analysis performed on genes with at least 10 reads. Normalization and differential expression analyses were performed using the DESeq2 Bioconductor^67^ package within the R statistical programming environment. Principal component analysis (PCA) was performed using the variance stabilizing transformation of DESeq normalized gene counts. False discovery rate (FDR) was calculated to control for multiple hypothesis testing of differential expression for pairwise comparisons. Genes expression changes with a base-2 log fold-change (LogFC) magnitude greater than one (|LogFC|> 1) and FDR adjusted p-value < 0.01 (padj < 0.01) were considered significant. The “Top 25 Expressed Genes” tables were created by identifying the most highly expressed genes by transcript count and filtering based on logFC > 1 and padj < 0.01 when compared to the second group. Gene set enrichment analysis (GSEA)^68,69^ was performed using the KEGG pathway geneset database^70^ comparing inner and outer zone meniscus tissue to passaged cells from corresponding zones. Genesets with FDR q-value < 0.25 that were commonly up- and down-regulated in both inner tissue versus passaged inner cells and outer zone tissue versus passaged outer cells were identified.

### Gene expression validation experiments

#### Sample collection

Cartilage (N = 13), meniscus tissue (N = 13/zone), and passaged meniscus cells (N = 3/zone) were isolated from 2–3 year old skeletally mature female pigs to validate the RNA- seq results. Meniscus tissue was harvested from the outer one-third and inner one-third of the medial menisci to definitively separate the inner and outer meniscal zones (Supplemental Fig. 2). All other harvest, culture, and RNA extraction procedures were performed as described above for both explants and cells.

#### Reverse transcription-quantitative polymerase chain reaction

RNA samples were reverse transcribed using the SuperScript VILO complementary DNA Synthesis Kit (Catalog # 11,754,050; Life Technologies). Quantitative polymerase chain reaction (qPCR) was performed using PowerUP SYBR Green Master Mix (Catalog # A25776; Thermo Fisher Scientific Baltics UAB) and the StepOne Plus Real-time PCR System (Model 4,376,374; Applied Biosystems). Supplemental Table 1 lists the porcine specific PCR primers for each target. Relative fold-change was determined by the 2^-∆∆Ct^ method^71^ using 18S as a reference gene and a single cartilage (Fig. 2) or outer zone meniscus tissue (Fig. 4) sample as the calibrator. There was no expression detected for *SNTB1* in 4 cartilage samples so a Ct of 40 (equivalent to the maximum number of PCR cycles) was assigned. Results are presented as log2 fold-change relative to the geomean of the indicated group. A Grubbs’ test was performed to identify outliers, which were subsequently removed. For data that was normally distributed, one-way ANOVAs with Tukey’s post-hoc analyses were performed. *LUM* expression was not normally distributed and thus a Kruskal–Wallis test with Dunn’s multiple comparison was performed. Groups were considered statistically significant at p < 0.05.

## Electronic supplementary material

Below is the link to the electronic supplementary material.

Supplementary Dataset 1

Supplementary Dataset 2

Supplementary Dataset 3

Supplementary Dataset 4

Supplementary Dataset 5

Supplementary Figures

## Data Availability

The datasets presented in this study can be found in the NCBI Gene Expression Omnibus (GEO) under accession numbers GSE275100.
