# Supplementary material for: Meniscus gene expression profiling of inner and outer zone meniscus tissue compared to cartilage and passaged monolayer meniscus cells
Source: Sci Rep. 2024 Nov 9;14:27423. doi: 10.1038/s41598-024-78580-3 (PMC11550462; doi:10.1038/s41598-024-78580-3)
Supplement: Supplementary file 6 — Supplementary Figures [file 41598_2024_78580_MOESM6_ESM.docx]

**Meniscus Gene Expression Profiling of Inner and Outer Zone Meniscus Tissue Compared to Cartilage and Passaged Monolayer Meniscus Cells**

Kaileen Fei^1^^, Benjamin D. Andress^1,2^^, A’nna M. Kelly^1,3^,

Dawn A. D. Chasse^1^, and Amy L. McNulty^1,2,3*^

Departments of ^1^Orthopaedic Surgery and ^2^Pathology, Duke University School of Medicine, Durham, NC, USA; ^3^Department of Biomedical Engineering, Duke University, Durham, NC, USA

^^^These authors have contributed equally to this work and share first authorship


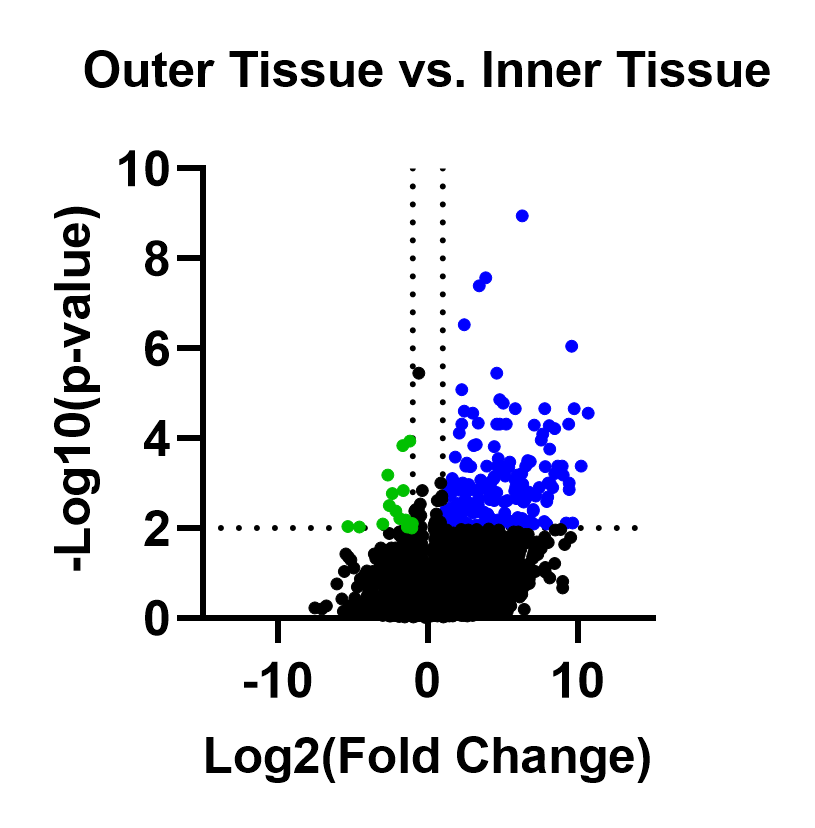


**Outer Meniscus vs Inner Meniscus**

**Supplemental Figure 1: Differentially Expressed Genes Between Outer and Inner Zone Meniscus Tissue.** Volcano plot showing genes significantly differentially expressed between outer and inner zone meniscus tissue. Each data point is an individual gene. Dashed lines indicate cutoffs for significant genes (FDR adjusted p-value < 0.01 and a |base-2 log fold-change| > 1). Color denotes if the gene was significantly up-regulated (blue) or down-regulated (green) in outer zone tissue relative to inner zone, or not significantly different (black).


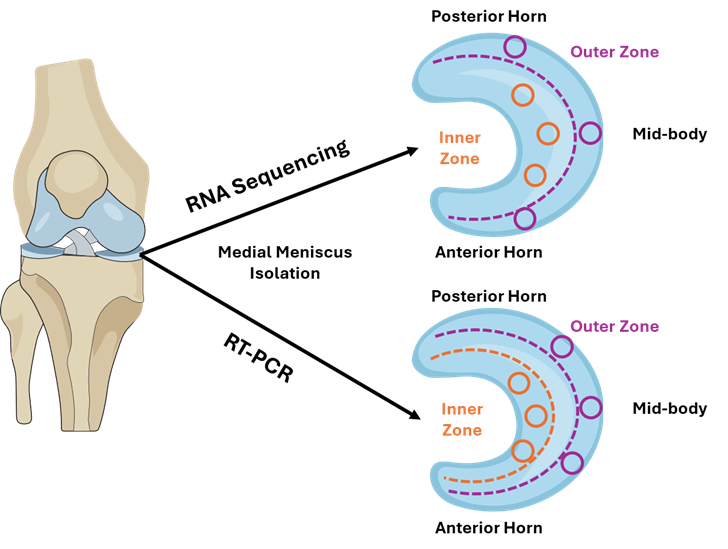


**Supplemental Figure 2: Schematic Depicting the Locations of Inner and Outer Zone Meniscus Tissue Explants.** Medial menisci were harvested from porcine knee joints and explants were harvested from both the inner (orange) and outer (purple) zones of the meniscus. For RNA-seq, explants were harvested from the outer one-third and inner two-thirds of the tissue. A total of three menisci (N=3) were used and a single explant was harvested for RNA-seq from each of the indicated positions (n=1 explant/zone/meniscus) to account for variability across the horns and midbody. For qRT-PCR validation, a total of 13 menisci (N=13) were used and explants were harvested from the outer one-third and the inner one-third of the tissue. The middle-third was discarded, assuring that RNA was not isolated from a mixed or transitional zone in the middle of the meniscus.

**Supplemental Table 1: qRT-PCR Porcine Primer Sequences**

| **Gene** | **Primer Pair (5' -> 3')** |
| --- | --- |
| ACTA2 | GCAATCAGGGGATGAGGACAT |
|  | GGTGCTTCACAGGGTCAGAT |
| AEBP1 | CCGATCCGGGACCATCAAT |
|  | ACTCTCGTGAGGGAACTTGTC |
| CILP2 | CAGGCCAACAGCTCCATATC |
|  | CAGGTAGGGCTTTTCCAACTT |
| FSTL1 | CCATGACCTGTGACGGAAAGA |
|  | GCTCCTGGACATATCTGGTCAT |
| LUM | AGCAGTGTCAAGACAGTAAGGATT |
|  | GATTGTCGCCAAGAGGAGAGT |
| PHLDA1 | CCTGGGCCAGACAAGGTTTTG |
|  | TGCGGACTGGGAGACTGTTT |
| PRRX1 | TACCTCGTCCTGCTCCAAGA |
|  | AGTAGCCATGGCGCTGTAA |
| TAGLN | GCCTGGGCTTCCAGATTTT |
|  | GTCCTCAGCGGCCTTTAAGA |
|  |  |
